# Supplementary figures and images for: Fluctuating selection across years and phenotypic variation in food-deceptive orchids
Source: PeerJ. 2017 Aug 25;5:e3704. doi: 10.7717/peerj.3704 (PMC5572944; doi:10.7717/peerj.3704)

A

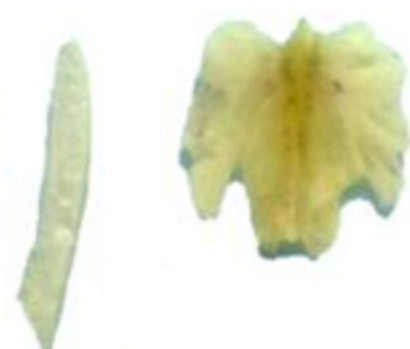

B

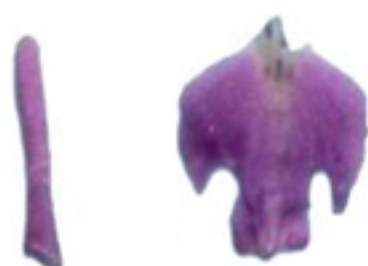

Supplement: Figure S1 — Scanned digital images of (A) O. pauciflora and (B) O. mascula floral parts. From left to right: spur, labellum. [file peerj-05-3704-s001.pdf]
